# Suggestive diagnosis of attention-deficit/hyperactivity disorder in indigenous children and adolescents from the Brazilian Amazon

**DOI:** 10.1007/s00787-019-01356-y

**Published:** 2019-06-04

**Authors:** Paulo Verlaine Borges e Azevêdo, Leonardo Ferreira Caixeta, Daniela Londe Rabelo Taveira, Margareth Rocha Peixoto Giglio, Maria Conceição do Rosário, Luis Augusto Rohde

**Affiliations:** 1 Professor of Psychiatry, School of Medicine, Pontifical Catholic University of Goiás (PUC-Goiás), Goiânia, GO Brazil; 2 School of Medicine, Federal University of Goiás (UFG), Goiânia, GO Brazil; 3 Professor of Gynaecology, School of Medicine, Pontifical Catholic University of Goiás (PUC-Goiás), Goiânia, GO Brazil; 4 Department of Psychiatry, Child and Adolescent Psychiatry Unit (UPIA), Federal University of São Paulo (UNIFESP), São Paulo, SP Brazil; 5 Child Study Center, Yale University, New Haven, CT USA; 6 Professor of Psychiatry, Medical School of Hospital de Clínicas de Porto Alegre, Federal University of Rio Grande Do Sul (UFRGS), Porto Alegre, RS Brazil; 7 National Institute of Developmental Psychiatry for Children and Adolescents, São Paulo, SP Brazil

**Keywords:** CBCL, TRF, ADHD, Indigenous, Children, Adolescents

## Abstract

The prevalence of attention-deficit/hyperactivity disorder (ADHD) symptoms has been scarcely studied in indigenous cultures that preserve ancestral cultural characteristics. The objective of the study is to estimate the prevalence of suggestive diagnosis of ADHD among indigenous children and adolescents from villages in the Amazon. This is an analytical cross-sectional study using instruments to track ADHD symptoms (the Child Behaviour Checklist for ages 6–18: CBCL/6–18 and the teacher report form for ages 6–18: TRF/6–18) and to investigate their negative impact on the patients (using the Strengths and Difficulties Questionnaire—SDQ). The prevalence of a suggestive ADHD diagnosis according to the CBCL/TRF DSM-IV ADHD subscale without and with negative impact as assessed by the SDQ was 4.3% and 1.1%, respectively. Comorbid oppositional-defiant, conduct problems and anxious symptoms were present in all cases screening positive for ADHD. We also presented a case report as an illustration of the observed clinical presentation. ADHD is a recognizable disorder even in a culture that preserves millennial characteristics. Furthermore, the presence of ADHD symptoms was associated with significant impairment.

## Introduction

Attention-deficit/hyperactivity disorder (ADHD) is a neurodevelopmental disorder, characterized by the presence of inattentive and/or hyperactive/impulsive symptoms disproportionate to the developmental stage, causing functional impairment in more than one environment [1, 2]. It afflicts approximately 5% of children and adolescents [3, 4] and 2.5% [1] to 4.4% [5] of adults. The disorder has severe consequences for the individual and the society, such as academic and professional impairment, increased risk for substance abuse and dependence, car accidents and criminality [1].

Despite having a well-established diagnosis and treatment, there are still discussions about its cross-cultural validity [6, 7]. It is questioned whether differences in worldwide prevalence would reflect changes in the incidence or tolerance of different societies to inappropriate behaviours [8]. Besides, little is known about its prevalence in ethnic minorities, such as indigenous people. Although there are few published studies on ADHD in these populations, some previous reports can be found in the United States [9–11], Canada [12], Australia [13] and Norway [14]. In Brazil, there has been only one previous observational study of indigenous children and adolescents with symptoms suggestive of ADHD [15].

Despite the relevance, none of these studies evaluated indigenous villages, with preserved millennial old cultural characteristics. To fill this gap, we investigated the prevalence of ADHD symptoms in a population of indigenous village children and adolescents. We hypothesized that the frequency of ADHD symptoms would be similar to that of other epidemiological studies, reinforcing the notion that ADHD does not result from cultural factors.

## Methods

### Study design

This was a cross-sectional and analytical epidemiological study.

### Ethical approval

Initially, we obtained the approval of the Research Ethics Committee of the Medical School at the Federal University of Goiás. Later we had authorization from the National Commission for Ethics in Research from the National Health Council of the Brazilian Ministry of Health. Finally, we obtained permission from the National Indian Foundation of the Ministry of Justice of Brazil (FUNAI/MJ). These steps followed detailed clarifications on the research project and consent of indigenous leaders. The entire process to obtain the ethical and legal releases for this study took 2 years.

### Population/sample

The population of indigenous children and adolescents studied belongs to the Karajá ethnic group and lives on the largest fluvial island on the planet. Bananal Island is 20,000 km^2^ and is located in the Araguaia River, a tributary of the Amazon Basin. They preserve their culture, passed down by their ancestors from almost 10,000 years ago. The Karajá is one of the 305 indigenous ethnic groups in Brazil. They are bilingual, speaking the native Karajá language and Portuguese, the official Brazilian language.

**Fig. 1:**
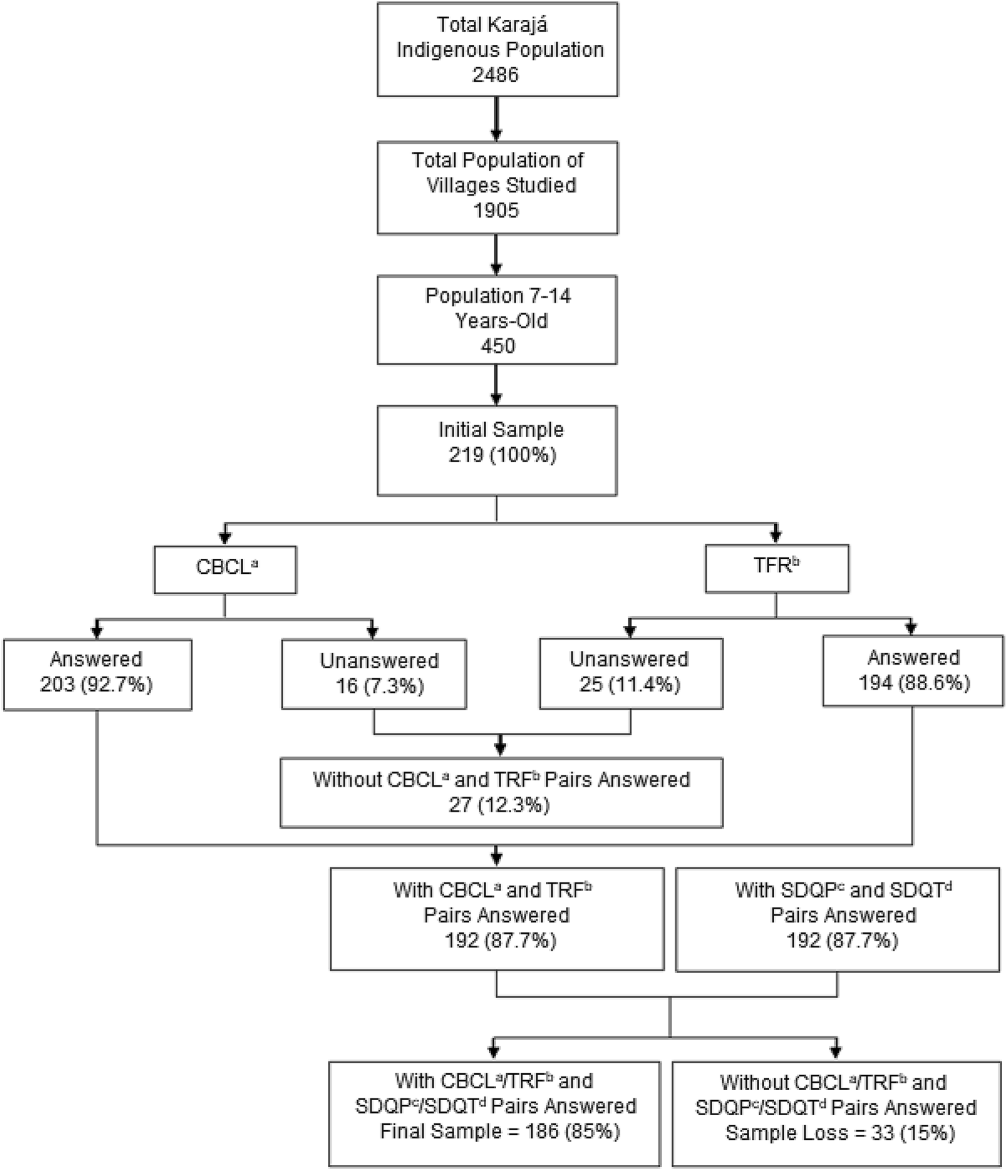
Sample flowchart of indigenous (Karajá) children and adolescents (7 to 14 years old), living in villages in the Brazilian Amazon Basin, in 2007. ^a^CBCL = *Child Behaviour Checklist Ages 6 to 18*-Brazilian version. ^b^TRF = *Teacher Report Form Ages 6 to 18*-Brazilian version. ^c^SDQP = *Strengths and Difficulties Questionnaire 4 to 17-*Brazilian version for parents or caregivers. ^d^SDQT = *Strengths and Difficulties Questionnaire 4 to 17-*Brazilian version for teachers

According to the 2007 census carried out by the National Health Foundation, the total indigenous population of this ethnic group was 2486. Of these, 1905 lived in the four villages participating in the current research. The number of individuals from 7 to 14 years of age was 450 (242 boys and 208 girls). The initial sample size calculated to assess the prevalence of ADHD in this population was 219, but at the end, 186 subjects participated in the study (Fig. 1). As a parameter, we adopted the average prevalence of 5.3% of ADHD in the world population of children and adolescents [4]. We used a 5% error estimate, with a 95% confidence interval.

The chosen villages were located relatively far from the urban centres, but easily accessible by the river. Tiny communities, those difficult to reach, and those very close to—or even in—urban centres, were excluded. We were looking for a sample that preserved, as much as possible, the fundamental cultural aspects of their ethnicity.

Children and adolescents who participated in the study were between 7 and 14 years of age and had at least one indigenous Karajá parent. The choice of this age group was made for two reasons: (1) to assess the mental health of children and adolescents, it is essential that at least two sources of information be researched (family and school). The Karajá children only start school at the age of 7 so younger children could not be included; (2) at the age of 14–15, the boys of this ethnic group participate in the ritual of passage to adult life, called *Hetohoky*. Girls at 14–15 years of age are already married and have children.

The subjects were chosen systematically, according to the list of names on the spread sheet of the 2007 population census: one participant was chosen and then the next (not alternately) until the stipulated number for the age group, sex and village was reached. The selected participant was excluded if the parents or guardians did not sign the informed consent form.

As presented in Table 1, of the 186 interviewees who were guardians of children/adolescents, 162 (87.1%) were biological parents, 2 (1.1%) were step-parents, 9 (4.8%) were grandparents and 13 (7%) were allocated into the category of “others” (i.e., foster parents). Of the biological parents that participated, 116 (62.4%) were mothers and 46 (24.7%) were fathers. Of the step-parents that participated, two (1.1%) were step-fathers. Participating grandparents were three (1.6%) grandmothers and six (3.2%) grandfathers, while seven (3.8%) of the “others” were women, and six (3.2%) were men.

**Table 1:** Distribution of interviewed guardians of the 186 indigenous children/adolescents

Guardians	Count (%) of children/adolescents in each grade									Total
	Preschool	1st	2nd	3rd	4th	5th	6th	7th	8th	
Biological mothers	7 (3.8)	26 (14.0)	29 (15.6)	20 (10.8)	12 (6.4)	12 (6.4)	9 (4.8)	0 (0.0)	1 (0.5)	116 (62.4)
Grandmothers	0 (0.0)	0 (0.0)	0 (0.0)	1 (0.5)	2 (1.1)	0 (0.0)	0 (0.0)	0 (0.0)	0 (0.0)	3 (1.6)
Other women	0 (0.0)	2 (1.1)	1 (0.5)	0 (0.0)	4 (3.2)	0 (0.0)	0 (0.0)	0 (0.0)	0 (0.0)	7 (3.8)
Total women	7 (3.8)	28 (15.0)	30 (16.1)	21 (11.3)	18 (9.7)	12 (6.4)	9 (4.8)	0 (0.0)	1 (0.5)	126 (67.8)
Biological fathers	1 (0.5)	6 (3.2)	13 (7.0)	6 (3.2)	6 (3.2)	4 (2.1)	5 (2.7)	3 (1.6)	2 (1.1)	46 (24.6)
Step-fathers	0 (0.0)	0 (0.0)	0 (0.0)	0 (0.0)	1 (0.5)	0 (0.0)	1 (0.5)	0 (0.0)	0 (0.0)	2 (1.1)
Grandfathers	0 (0.0)	2 (1.1)	1 (0.5)	1 (0.5)	0 (0.0)	1 (0.5)	0 (0.0)	1 (0.5)	0 (0.0)	6 (3.2)
Other men	0 (0.0)	3 (1.6)	2 (1.1)	0 (0.0)	0 (0.0)	1 (0.5)	0 (0.0)	0 (0.0)	0 (0.0)	6 (3.2)
Total men	1 (0.5)	11 (5.9)	16 (8.6)	7 (3.8)	7 (3.8)	6 (3.2)	6 (3.2)	4 (2.1)	2 (1.1)	60 (32.2)
Total	8 (4.3)	39 (21.0)	46 (24.7)	28 (15.0)	25 (13.4)	18 (9.7)	15 (8.1)	4 (2.1)	3 (1.6)	186 (100.0)

The number of children/adolescents according to schooling and to the person who answered the CBCL (biological parents, grandparents, step-parents and others—foster parents) are presented here

Forty teachers (25 indigenous and 15 non-indigenous) participated, including ten women (8 indigenous and 2 non-indigenous) and 30 men (17 indigenous and 13 non-indigenous). The 10 women answered screening questions regarding 48 (25.8%) children/adolescents and the 30 men provided qualitative information about 138 (74.2%) children/adolescents. Indigenous teachers only taught up to the fourth grade, and non-indigenous teachers taught between the fifth and eighth grades. This is because a college degree is mandatory for those teaching from the fifth to eighth grade, and only non-indigenous teachers had such prerequisites. The complete distribution of children/adolescents among participating teachers is presented in Table 2.

**Table 2:** Distribution of indigenous children/adolescents for each teacher interviewed according to sex and ethnicity

Tribe	Teacher ethnicity	Teacher sex	Count (%) of children/adolescents in each grade									Total
			Preschool	1st	2nd	3rd	4th	5th	6th	7th	8th	
1	Indigenous	Women	0 (0.0)	7 (3.8)	0 (0.0)	0 (0.0)	3 (1.6)	0 (0.0)	0 (0.0)	0 (0.0)	0 (0.0)	10 (5.4)
		Men	1 (0.5)	6 (3.2)	19 (10.2)	9 (4.8)	7 (3.8)	0 (0.0)	0 (0.0)	0 (0.0)	0 (0.0)	42 (22.6)
	Non-indigenous	Women	0 (0.0)	0 (0.0)	0 (0.0)	0 (0.0)	0 (0.0)	0 (0.0)	1 (0.5)	0 (0.0)	0 (0.0)	1 (0.5)
		Men	0 (0.0)	0 (0.0)	0 (0.0)	0 (0.0)	0 (0.0)	2 (1.1)	1 (0.5)	1 (0.5)	1 (0.5)	5 (2.7)
2	Indigenous	Women	6 (3.2)	0 (0.0)	0 (0.0)	10 (5.4)	0 (0.0)	0 (0.0)	0 (0.0)	0 (0.0)	0 (0.0)	16 (8.6)
		Men	0 (0.0)	12 (6.5)	11 (5.9)	1 (0.5)	7 (3.8)	0 (0.0)	0 (0.0)	0 (0.0)	0 (0.0)	31 (16.7)
	Non-indigenous	Women	0 (0.0)	0 (0.0)	0 (0.0)	0 (0.0)	0 (0.0)	1 (0.5)	0 (0.0)	0 (0.0)	0 (0.0)	1 (0.5)
		Men	0 (0.0)	0 (0.0)	0 (0.0)	0 (0.0)	0 (0.0)	4 (2.2)	10 (5.4)	0 (0.0)	0 (0.0)	14 (7.5)
3	Indigenous	Women	0 (0.0)	0 (0.0)	10 (5.4)	0 (0.0)	0 (0.0)	0 (0.0)	0 (0.0)	0 (0.0)	0 (0.0)	10 (5.4)
		Men	1 (0.5)	7 (3.8)	2 (1.1)	4 (2.2)	7 (3.8)	0 (0.0)	0 (0.0)	0 (0.0)	0 (0.0)	21 (11.3)
	Non-indigenous	Men	0 (0.0)	0 (0.0)	0 (0.0)	0 (0.0)	0 (0.0)	9 (4.8)	2 (1.1)	3 (1.6)	1 (0.5)	15 (2.7)
4	Indigenous	Women	0 (0.0)	5 (2.7)	1 (0.5)	4 (2.2)	0 (0.0)	0 (0.0)	0 (0.0)	0 (0.0)	0 (0.0)	10 (5.4)
		Men	0 (0.0)	2 (1.1)	3 (1.6)	0 (0.0)	1 (0.5)	0 (0.0)	0 (0.0)	0 (0.0)	0 (0.0)	6 (3.2)
	Non-indigenous	Men	0 (0.0)	0 (0.0)	0 (0.0)	0 (0.0)	0 (0.0)	2 (1.1)	1 (0.5)	0 (0.0)	1 (0.5)	4 (2.2)
All	Indigenous	Women	6 (3.2)	12 (6.5)	11 (5.9)	14 (7.5)	3 (1.6)	0 (0.0)	0 (0.0)	0 (0.0)	0 (0.0)	46 (24,8)
	Non-Indigenous		0 (0.0)	0 (0.0)	0 (0.0)	0 (0.0)	0 (0.0)	1 (0.5)	1 (0.5)	0 (0.0)	0 (0.0)	2 (1.0)
	Total		6 (3.2)	12 (6.5)	11 (5.9)	14 (7.5)	3 (1.6)	1 (0.5)	1 (0.5)	0 (0.0)	0 (0.0)	48 (25.8)
All	Indigenous	Men	2 (1.1)	27 (14.5)	35 (18.8)	14 (7.5)	22 (11.8)	0 (0.0)	0 (0.0)	0 (0.0)	0 (0.0)	100 (53.8)
	Non-Indigenous		0 (0.0)	0 (0.0)	0 (0.0)	0 (0.0)	0 (0.0)	17 (9.1)	14 (7.5)	4 (2.2)	3 (1.6)	38 (20.4)
	Total		2 (1.1)	27 (14.5)	35 (18.8)	14 (7.5)	22 (11.8)	17 (9.1)	14 (7.5)	4 (2.2)	3 (1.6)	138 (74.2)
All	Ethnicities	Women	6 (3.2)	12 (6.5)	11 (5.9)	14 (7.5)	3 (1.6)	1 (0.5)	1 (0.5)	0 (0.0)	0 (0.0)	48 (25.8)
		Men	2 (1.1)	27 (14.5)	35 (18.8)	14 (7.5)	22 (11.8)	17 (9.1)	14 (7.5)	4 (2.2)	3 (1.6)	138 (74.2)
		Total	8 (4.3)	39 (21.0)	46 (24.7)	28 (15.1)	25 (13.4)	18 (9.7)	15 (8.1)	4 (2.2)	3 (1.6)	186 (100.0)

This table has been distributed according to sex (woman and man) and ethnicity (indigenous and non-indigenous) of the teacher. Data is present for each tribe, compares ethnicity and sex (woman and man), and identifies totals for each sex, regardless of ethnicity

The parents or guardians and teachers of the participating children and adolescents signed informed consent forms. The study was explained in detail to children and adolescents before they assented to participating.

### Instruments

The Achenbach System of Empirically Based Assessment (ASEBA) [16–18] was used in this study: The Child Behaviour Checklist for Ages 6–18 (CBCL/6–18) was applied to parents or guardians and the Teacher Report Form for Ages 6–18 was answered by teachers (TRF/6–18). Although the instruments have validated Brazilian versions [19, 20] and are quite sensitive to probable cases of mental problems [21], there was no validation in the indigenous population.

Although not designed as diagnostic tools for ADHD, they have a high sensitivity to detect individuals at risk and probable comorbidities [22]. Also, they are excellent instruments for non-clinical population samples. Finally, there is an excellent convergence between the attention deficit scale and the diagnosis of ADHD assigned through structured interviews [23, 24].

The questionnaires recorded the behavioural and emotional problems of children and adolescents. Scores were obtained for (a) 118 items of specific problems and two items of open/closed questions; (b) the eight-syndrome scales of the CBCL: anxious/depressed, withdrawn/depressed, somatic complaints, social problems, thought problems, attention problems, rule-breaking behaviour, and aggressive behavior; (c) two scales derived from the eight syndrome scales, termed internalizing problems (anxious/depressed, withdrawn/depressed, and somatic complaints) and externalizing (rule-breaking behavior and aggressive behaviour); and (d) total problems, consisting of the sum of the scores of the 118 problem items [16, 19].

Also, the questionnaires presented six probable diagnoses compatible with the Diagnostic and Statistical Manual of Mental Disorders*-*IV (DSM-IV). These are named affective problems, anxiety problems, somatic problems, attention deficit/hyperactivity problems, oppositional defiant problems and conduct problems [16].

Each question had three possible answers (0 = not true, 1 = sometimes true and 2 = often true). The questions referred to the last 6 and 2 months of life, in the CBCL and TRF, respectively. The cut-off points of the sum of the scores provided three categories: clinical (> 63), borderline (≥ 60 and ≤ 63) and nonclinical (< 60) [19].

The positive predictive value is high in the tracking of behavioral and emotional problems of children and adolescents of different cultures [17, 25]. They are the most widely used and accepted instruments worldwide [18]. Finally, they are easily manageable, self-administered or administered by lay interviewers [26].

For this study, we used the data equivalent to the DSM-IV diagnoses compatible with the CBCL and TRF scales-syndromes. Thus, the results were expressed as positive screening for affective problems (AffD), anxiety problems (AnxD), attention-deficit/hyperactivity problems (ADHD), oppositional defiant problems (ODD), and conduct problems (CD).

The diagnostic criteria for ADHD from the DSM-IV included in both the CBCL and TRF were fails to finish the activities; cannot concentrate; cannot sit still; fidgets; impulsive; inattentive; talks much and loud. For the TRF: difficulty with directions; disturbs others; talks out of turn; disrupts class; fails to carry out tasks were also present [16].

We proceeded with additional (semantic and cultural) adaptation of the CBCL and TRF from Portuguese to Karajá, because there were rarely exact matches between them. We tried to use instruments that were as attentive to the particularities of that culture as possible. This adaptation process was authorized by Dr. Thomas M. Achenbach, author of ASEBA.

Additional culturally appropriate procedures occurred at various times. Initially, individuals from different social levels of the ethnic group were interviewed. They included leaders, indigenous officials in the service of National Health Foundation (FUNASA/MS) and the National Indian Foundation of the Ministry of Justice of Brazil (FUNAI/MJ), as well as individuals from the general community. At this stage, some Portuguese terms were replaced with Karajá, to increase understanding of the issues. Then we performed three pre-tests and new adjustments to reach the maximum knowledge of the problems of the indigenous community.

The Strengths and Difficulties Questionnaire (SDQ) [27–29] impact supplement was used to assess the impairment caused by the symptoms, applied to parents or guardians, and teachers of children and adolescents. This instrument was validated for Brazilian Portuguese by Fleitlich-Bilyk and Goodman [30]. It has good validity and reliability for rich, poor, rural and urban populations, [31, 32].

The Portuguese version consists of SDQ Pa^4−17^ and SDQ Pr^4−17^, respectively, for parents or caregivers, and teachers of young people aged 4–17 years. Impact supplements assess the suffering and dysfunction in the personal, academic and socio-familial spheres [27, 33, 34]. The supplement consists of five questions with three possible scores: 0 = normal, 1 = borderline and 2 = changed. The total score ranges from 0 to 10 (0 = considered normal, 1 = borderline and ≥ 2 = altered).

The literature has demonstrated a disagreement between information provided by the parents/caregivers and the children’s teachers [14, 16, 35, 36]. Therefore, it is extremely relevant to collect and analyse data from both types of informants. For instance, one study assessing parent–teacher agreement of the SDQ found that child's gender and age, mother’s employment status, single-parent home, and the number of children in the household were relevant factors of informant disagreement [35]. In another study, caregiver reports of attention problems were more useful than teacher reports and self-reports in identifying ADHD, and combining caregiver and teacher reports improved the ADHD identification [36]. Therefore, it is important to gather data from multiple informants, and when both parents/legal guardians and teachers agree with one another, there is a much higher probability of having a true ADHD diagnosis.

### Data collection and analysis

Information was collected between July 2007 and November 2008. Only one (non-native) interviewer, a nurse responsible for the special indigenous health district (DSEI), applied the questionnaires. In this way, there was no risk of variation by more than one interviewer. This professional was the person best accepted by the Karajá, as they were already used to her presence in the village. She was trained by the lead author of this study before conducting the interviews. The questionnaires were applied first to teachers and then to parents or caregivers. We chose the direct interview method because many of the parents or guardians were illiterate. Teachers were also interviewed to standardize the collection.

The data collected were stored and analysed by the Statistical Package for Social Science version 18.0 (SPSS 18.0). In the descriptive evaluation, we studied the variables of age, sex, and schooling. The age variable was divided into two categories (7–10 and 11–14 years of age). The variable education was divided between grades 1–4 and grades 5–8 of elementary school.

The prevalence of signs and symptoms of ADHD and comorbidities was estimated for the results compatible with such diagnoses by DSM-IV in CBCL and TRF. For the analysis of the gender, age, and schooling variables, we used two categories: nonclinical (normal and borderline) and clinical (positive triage cases). We chose such categories to reduce false positives, which would be more likely if we compared normal with altered (borderline and clinical). We analysed the answers from parents or guardians (CBCL) and teachers (TRF) separately. Finally, we investigated the combined responses of the interviewees, considering as positive those cases for which both information sources agreed (CBCL and TRF).

We initially analysed the results without considering the negative impact of the problems and then analysed them considering the negative impact. In the first situation, we observed the prevalence estimated by the positive cases for ADHD, according to the CBCL and TRF individually and in combination. In the second situation, we considered only the triage cases with the SDQ supplement indicating the negative impact. Regarding the negative impact, we considered as positive those cases with positive scores by both parents/caregivers and teachers.

A combination of assessment strengths from the ASEBA and SDQ screening instruments was used, including both parents/legal guardians and teachers as information sources. Where the ASEBA has a more detailed list of symptoms [16], the SDQ has a broader ability to assess the suffering and dysfunction in the personal, academic and socio-familial domains of the individual [27, 28].

Finally, we presented a case report to illustrate clinical presentations of symptoms observed during an interview, in the context of the health service delivered to the population on their tribes.

The chi-square test (*χ*^2^) was used for making comparisons of categorical variables between the groups. When the samples were too small, we used Fisher’s exact test. The Kappa index (κ) was used to assess the level of agreement between respondents (parents or caregivers vs. teachers) regarding the symptoms of ADHD. We also evaluated the concordance (κ) between the corresponding trait (CBCL, TRF) and impact (SDQPa^4−17^, SDQPr^4−17^). The level of significance was 5% (*p* < 0.05), with a 95% confidence interval (CI).

## Results

The response rate for this study was 85% (Fig. 1). The sample loss (15%) is explained by participants moving during the time of the study (10%) and participant drop out (5%). Those who dropped out of the study did so as a result of interview fatigue. In such cases, we eliminated the participant as a result of incomplete information (5%).

The sample finally analysed had 186 subjects (Fig. 1), 85 (45.7%) girls and 101 (54.3%) boys. The group was equally divided between the two age groups, 93 (50%) were 7–10 years of age, and 93 (50%) were 11–14 years of age. Up to and including fourth grade, there were 146 (78.5%) subjects and 40 (21.5%) subjects in fifth to eighth grade.

**Fig. 2:**
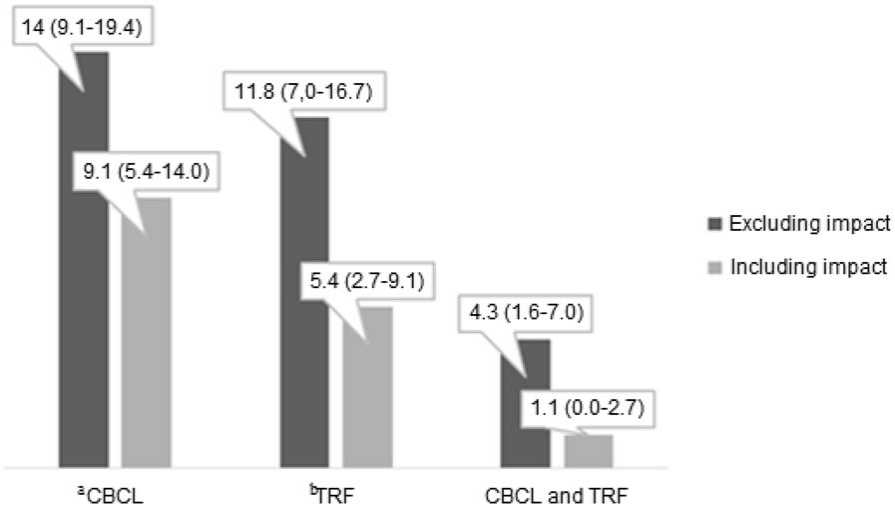
The estimated prevalence of ADHD symptoms (according to DSM-IV) found in the sample (N = 186) of Karajá children and adolescents (7–14 years old) in the Amazon Basin. Investigation through parents/caregivers (CBCL, *n* = 26 without and *n* = 17 with functional impact), teachers (TRF, *n* = 22 without and *n* = 10 with functional impact), and through both combined (CBCL and TRF, *n* = 8 without and *n* = 2 with functional impact). The percentages (%) and the 95% CI are presented at the top of each bar. ^a^CBCL—*Child Behaviour Checklist*/6 *to* 18 (*Brazilian version* ); ^b^TRF—*Teacher Report Form*/6 *to* 18 (*Brazilian version*)

The estimated rates of positive screening for ADHD were 4.3% (CI95% 1.6–7.5) without and 1.1% (CI95% 0.0–2.7) with functional impact (Fig. 2). The agreement between the responders (CBCL vs. TRF) was low, both disregarding (κ = 0.235) and considering (κ = 0.086) the impact. The concordance rate between the responses to the screening instruments and their impact supplements was good (κ = 0.765 for parents or caregivers and κ = 0.595 for teachers). The Kappa index between the combined results (CBCL and TRF), considering or not considering the negative impact, was moderate (κ = 0.389).

Eight participants (8/186) had positive ADHD screening by both responders (CBCL and TRF), without considering the functional impact (Fig. 2). Considering the negative impact, two participants (2/186) had a positive screening for ADHD (Fig. 2). There was a statistical difference only for the teachers’ responses (TRF). The prevalence was higher in younger children (*p* = 0.006) and the early school years (*p* = 0.039) (Table 3), disregarding the negative impact. The rate was significant only for younger children (*p* = 0.009) (Table 3), considering the negative impact.

**Table 3:** Comparisons across the estimated prevalence of ADHD symptoms found through CBCL (parents or surrogates) and TRF (teachers) individually made and grouped (CBCL and TRF)

Variable	Prevalence (%) 95% CI														
	CBCL^a^					TRF^b^					CBCL^a^ and TRF^b^				
	No impact	*p*	Impact	*p*	κ	No impact	*p*	Impact	*p*	κ	No impact	*p*	Impact	*p*	κ
Gender															
Female	7.0 3.8–11.3	0.635	4.8 2.2–8.1	0.529	0.807	4.3 1.6–7.5	0.349	1.6 0.0–3.8	0.306	0.535	1.6 0.0–3.8	0.634	1.1 0.0–2.7	0.121	0.797
Male	7.0 3.2–10.8		4.3 1.6–7.0		0.749	7.5 3.8–11.8		3.8 1.1–7.0		0.649	2.7 0.5–5.4		0.0 NA		NA
Age															
7 to 10	8.6 5.4–12.9	0.205	5.4 2.2–8.6	0.445	0.753	9.1 5.4–13.4	0.006*	4.8 2.2–8.1	0.009*	0.672	3.2 1.1–5.9	0.148	1.1 0.0–2.7	0.155	0.492
11 to 14	5.4 2.2–9.1		3.8 1.1–6.5		0.815	2.7 0.5–5.4		0.5 0.0–1.6		0.327	1.1 0.0–2.7		0.0 NA		NA
Scooling															
Until 4th	12.4 8.1–17.2	0.182	8.1 4.3–12.4	0.305	0.767	11.3 7.0–16.1	0.039*	5.4 2.7–9.1	0.089	0.617	4.3 1.6–7.5	0.130	1.1 0.0–2.7	0.457	0.389
5th to 8th	1.6 0.0–3.7		1.1 0.5–2.7		0.797	0.5 0.0–1.6		0.0 NA		NA	0.0 NA		0.0 NA		NA

The table shows the results according to gender (female and male), age band (7–10 and 11–14 years old) and schooling ( ≤ Grade 4 and Grades 5–8). We considered all the positive screening (clinical) cases without (no impact) and with the functional impact (impact). We used the Kappa index (κ) to measure the intra-interviewed agreement on symptom tracing and negative effects and the concordance between the interviewers. The indigenous children and adolescents were living in tribes in the Brazilian Amazon (N = 186)

^a^CBCL – *Child Behaviour Checklist*/6 *to* 18 (*Brazilian version* ); ^b^TRF – *Teacher Report Form*/6 *to* 18 (*Brazilian version*); (NA) – *Not Applicable* ; *p < 0.05

We found ADHD comorbidity with ODD, CD, and AnxD in 100% of the cases with dysfunction (2/186). In the general population studied, the estimated prevalence of the same disorders were 2.7% for ODD (95% CI 0.5–5.4), 3.2% for CD (95% CI 1.1–5.9) and 2.7% for AnxD (95% CI 0.5–5.4). There was a significant difference in comorbidity rates (*p* < 0.001) among the positive screening cases for ADHD and the general population.

Disregarding the negative impact of the problems, we still observed high comorbidity rates with ADHD. Of the participants who screened positive for ADHD, 87.5% also screened positive for ODD and 75% for CD. Symptoms of anxiety were present in 62.5% and affective problems in 12.5% of the cases that screened positive for ADHD.

### Case report

We studied the prevalence of signs and symptoms indicative of ADHD, motivated by the demand of the indigenous community itself. The mother of a 10-year-old boy sought the responsible researcher Dr. Paulo Verlaine Borges e Azevêdo at the indigenous health post. She reported that her son was restless and his behaviour was continually getting in the way of his schooling and social cohabitation. He was also extremely inattentive, impulsive and was putting his own life at risk. Several times he would leave the classroom and swim across the Araguaia River, causing the village to worry about him.

The child was examined considering the values and expectations of the Karajá culture. The Karajá child is free to do virtually anything he wants until he/she is 7 years old. He/she enters the school at this age, and he/she is literate in the native language and only then learns Portuguese. During class, children can go out to bathe in the river and return later. To an unsuspecting non-indigenous observer, almost all Karajá children—disruptive and noisy during class—would be labelled as having ADHD. However, in their culture, this is allowed and does not prevent education from taking place according to the established curriculum.

The boy’s neuropsychomotor development was normal. The agitation had always been present, as had his inattention and impulsiveness. There were no other emotional changes, such as mood swings or anxious symptoms. In the examination, he did not show evidence of mental deficiency or psychotic symptoms (also culturally contextualized). The clinical diagnosis made by the child psychiatrist was ADHD, according to DSM-IV criteria [37]. Other families spontaneously sought the psychiatrist, bringing children who were agitated, impulsive, inattentive and disobedient, for evaluation and treatment [15].

How, in a culture that allows children total freedom, are parents bothered by ADHD-like behaviour? We observed that their behaviours extrapolated from those usually accepted. They hindered the life of the young person, the family, and the community, causing suffering and dysfunction. The first similarity between this community and other cultures was the search for help for disruptive behaviour in children. These were the main reasons for medical care in indigenous health posts: concerning mental health problems [15].

## Discussion

The indigenous population is one of the most unassisted in Brazil and in the world [38]. To our knowledge, this is the first study to systematically investigate ADHD symptoms in Brazilian indigenous children and adolescents [15]. We used validated and globally recognized screening instruments [19, 21] and well-trained interviewers to assess the sample. The results suggest a prevalence of ADHD that is similar to global prevalence. We discuss the implications of these findings, suggesting that an ADHD diagnosis may exist even in isolated cultures.

Rohde et al. [7] performed a systematic review of the literature on ADHD in Brazil and compared these findings to those from studies in developed countries, and found that ADHD is not a cultural construct. They also reinforced the importance of applying a similar research methodology between cultures to make findings comparable [7]. Polanczyk et al. [4] found that geographic location plays a limited role in the variability of worldwide ADHD prevalence estimates. Instead, such variability seems to be explained primarily by the methodological characteristics of studies [4]. In our research, a comparable research methodology was used when observing previous studies, and as a result, we believe in the assertion that ADHD is not a cultural construct.

Although not designed as a diagnostic tool, the ASEBA inventories show high agreement with DSM-IV criteria for ADHD [6, 18, 39]. Information was collected from parents or caregivers, and teachers. Different sources of information are fundamental to the psychopathological clinical evaluation of children and adolescents [17]. The perceptions of teachers and parents may differ more in indigenous populations than for most children because of different cultural norms [14]. As extensively documented in previous studies [14, 16, 35, 36], we found a low correlation between the answers of parents or guardians and teachers.

The question of cultural interpretation applies both to the differences between the various ethnic groups and to those between informants about the same child [14]. We emphasize that, even in the indigenous culture, the difference between parents and teachers in the perception of ADHD symptoms was remarkable.

Javo et al. [14] observed that indigenous Sami mothers mentioned fewer attention problems than mothers of other Norwegian children. Conversely, there were no differences between the two ethnicities when the teachers provided the information. An essential difference between the Sami and Karajá indigenous populations is in their parents’ schooling. The percentage of the Sami population who attended college even outnumbered Norwegians (63% vs. 52%) [14]. In our sample, most parents or guardians were illiterate.

On the other hand, the Sami are illiterate in their language [14], while the Karajá are literate in their native language. Sami children study in the same schools and have the same social and cultural norms as their Norwegian counterparts [14]. Karajá children and adolescents study in schools in their villages, predominantly with native teachers and a curriculum adapted to their reality. Thus, we believe that the Karajá culture is comparatively more preserved than the Sami culture.

The Norwegian versions of the CBCL and TRF could have problems of Sami–Norwegian equivalence [14]. We believe that the cultural adaptation of the instruments to Portuguese–Karajá minimized such risk.

As in our sample, Javo et al. [14] found a low correlation between the CBCL and the SDQ impact scores. These findings may be secondary to the lack of an instrument that simultaneously assesses both presence of symptoms and their impact on the subject’s life [18].

Like Javo et al., we used the CBCL and TRF for symptom screening and the respective SDQ supplement (Pa^4−17^ and Pr^4−17^) to assess functional impairment. The concordance rate between positive symptoms and negative impact was good in our study. However, there was a low agreement in the combination of answers from parents or caregivers and teachers.

The rates of ADHD varied widely in other populations of Brazilian children and adolescents from other regions of the country. The 1.1% rate found, considering the negative impact on children’s lives in more than one setting, would be quite conservative. However, it is important to note that Fleitlich-Bilyk and Goodman [32], using the Development and Well-Being Assessment (DAWBA), detected ADHD in 1.8% of children aged 7–14 in the interior of São Paulo (southeast region). Goodman et al. [40] detected 0.9% in children aged 5–14, in the northeast region, also using the DAWBA.

The ADHD prevalence in our sample was 4.3%, which is closer to the world average of 5.3%, disregarding the functional impact [4] and to the rate of 5.8% found by Rohde et al. [41] in adolescents (12–14 years old) from southern Brazil.

Two studies in the southeastern region of the country found rates much higher than ours. Vasconcelos et al. [42] reported a prevalence of 17.1% in a population aged 6–15, with a diagnostic evaluation. Fontana et al. [43] found symptoms in 13% of a sample of 6–12-year-olds in public schools. In another study in the southern region, Guardiola et al. [44] found ADHD symptoms in 17.9% of children in the first grade of elementary school, according to DSM-IV criteria. It is important to note that the high rates presented by the aforementioned studies are due to a lack of consideration for the functional impact of the symptoms by the authors, which was in fact considered in the current study. Another reason for these high rates is that these studies assessed only one source of informants (i.e., either teachers or parents).

Anselmi et al. [45], in a cohort in the south of the country, found ADHD symptoms in 4.2% and 2.7% of the sample, according to DSM-IV and ICD-10 criteria. They interviewed the parents in a screening phase and, subsequently, parents and pre-adolescents (4–11 years of age).

The rates of ADHD among Canadian indigenous children [12], as recorded by the Conners’ Parent and Teacher Revised Scales Revised-Long Version (CRS-R: L), were much higher (22.7%). The Canadian study used only a screening instrument and did not require impairment. In American-Indian children aged 9, 11 and 13 years, assessed with the Child and Adolescent Psychiatric Assessment (CAPA), the ADHD prevalence was 1.2% [11].

The ADHD prevalence tends to be higher in schools than in the community, as found in two Brazilian studies of teachers [42–44]. Vasconcelos et al. [42] and Guardiola et al. [44] found higher rates than ours, which were collected from two sources of information (home and school). Teachers indicated a higher prevalence of symptoms in younger children (7–10 years of age) and up to grade 4, consistent with the literature.

In contrast to studies of other cultures [4, 41–43], we did not find significant differences between boys and girls [10], both by CBCL and by TRF. This is most likely because of our sample size.

The rates of comorbid symptoms outweighed those commonly found for disruptive (oppositional-defiant and conduct) disorders of 30–50% [46]. The rate of affective symptoms (12.5%) is close to those reported in the international literature (15–20%) [46]. The symptoms of anxiety were more prevalent than those indicated in the literature (25%) [46].

One hypothesis for the high rates of comorbidities would be the fact that the Karajá tolerate the milder symptoms of ADHD. Therefore, those who were identified with ADHD symptoms would correspond to more severe cases and, thus, with higher comorbidities rates.

As limitations of our study, we can cite the fact that the sample was systematically allocated. The sample loss (15%) could also represent a selection bias since due to constant village changes and dropouts, this group might have a higher hyperactive component. Although all the caregivers (biological or adoptive parents or grandparents) were indigenous, there were indigenous (majority) and non-indigenous teachers interviewed. Perhaps there is a bias in the precise indication of what would be culturally accepted, or not, by non-indigenous teachers. Another limitation was the fact that we did not use diagnostic interviews. Despite these limitations, the current study included a significant sample of the population studied and a highly qualified professional conducted direct interviews with parents and teachers.

We conclude that ADHD symptoms are prevalent among Karajá children and adolescents and that they impact and impair their lives. The occurrence of symptoms suggestive of ADHD among the Karajá was similar to that of countless other cultures. Therefore, we believe that the nature component of the disorder becomes apparent, although modulated by the nurture.

From these findings, the need for studies evaluating the treatment of these children and adolescents becomes urgent. These results also reinforce the hypothesis that ADHD is not a consequence of cultural factors alone and that the nature component of the disorder becomes apparent, although modulated by the nurture.

## References

[1] Faraone SV, Asherson P, Banaschewski T, Biederman J, Buitelaar JK, Ramos-Quiroga JA (2015). Attention-deficit/hyperactivity disorder. Nat Rev Dis Primers.

[2] American Psychiatric Association (2013). Diagnosis and statistics manual of mental disorders.

[3] Polanczyk GV, Willcutt EG, Salum GA, Kieling C, Rohde LA (2014). ADHD prevalence estimates across three decades: an updated systematic review and meta-regression analysis. Int J Epidemiol.

[4] Polanczyk G, de Lima MS, Horta BL, Biederman J, Rohde LA (2007). The worldwide prevalence of ADHD: a systematic review and metaregression analysis. Am J Psychiatry.

[5] Polanczyk G, Rohde LA (2007). Epidemiology of attention-deficit/hyperactivity disorder across the lifespan. Curr Opin Psychiatry.

[6] Roessner V, Becker A, Rothenberger A, Rohde LA, Banaschewski T (2007). A cross-cultural comparison between samples of Brazilian and German children with ADHD/HD using the Child Behavior Checklist. Eur Arch Psychiatry Clin Neurosci.

[7] Rohde LA, Szobot C, Polanczyk G, Schmitz M, Martins S, Tramontina S (2005). Attention-deficit/hyperactivity disorder in a diverse culture: do research and clinical findings support the notion of a cultural construct for the disorder?. Biol Psychiatry.

[8] Timimi S, Taylor E (2004). ADHD is best understood as a cultural construct. Br J Psychiatry.

[9] Nasir BF, Hides L, Kisely S, Ranmuthugala G, Nicholson GC, Black E (2016). The need for a culturally-tailored gatekeeper training intervention program in preventing suicide among indigenous peoples: a systematic review. BMC Psychiatry.

[10] Lefler EK, Hartung CM, Bartgis J, Thomas DG (2015). ADHD Symptoms in American Indian/Alaska native boys and girls. Am Indian Alsk Native Mental Health Res..

[11] Costello EJ, Farmer EM, Angold A, Burns BJ, Erkanli A (1997). Psychiatric disorders among American Indian and white youth in Appalachia: the Great Smoky Mountains Study. Am J Public Health.

[12] Baydala L, Sherman J, Rasmussen C, Wikman E, Janzen H (2006). ADHD characteristics in Canadian Aboriginal children. J Atten Disord.

[13] Loh PR, Hayden G, Vicary D, Mancini V, Martin N, Piek JP (2016). Australian Aboriginal perspectives of attention deficit hyperactivity disorder. Aust N Z J Psychiatry.

[14] Javo C, Ronning JA, Handegard BH, Rudmin FW (2009). Cross-informant correlations on social competence and behavioral problems in Sami and Norwegian preadolescents. Eur Child Adolesc Psychiatry.

[15] Azevedo PV, Caixeta L, Andrade LH, Bordin IA (2010). Attention deficit/hyperactivity disorder symptoms in indigenous children from the Brazilian Amazon. Arq Neuropsiquiatr.

[16] Achenbach TM, Rescorla LM (2001). Manual for the ASEBA school-age forms and profiles.

[17] Achenbach TM (2010). Multicultural evidence-based assessment of child and adolescent psychopathology. Transcult Psychiatry.

[18] Achenbach TM, Becker A, Dopfner M, Heiervang E, Roessner V, Steinhausen HC (2008). Multicultural assessment of child and adolescent psychopathology with ASEBA and SDQ instruments: research findings, applications, and future directions. J Child Psychol Psychiatry.

[19] Bordin IA, Rocha MM, Paula CS, Teixeira MC, Achenbach TM, Rescorla LA (2013). Child Behavior Checklist (CBCL), Youth Self-Report (YSR) and Teacher’s Report Form (TRF): an overview of the development of the original and Brazilian versions. Cad Saude Publica.

[20] Rocha MM, Rescorla LA, Emerich DR, Silvares EF, Borsa JC, Araujo LG (2013). Behavioural/emotional problems in Brazilian children: findings from parents’ reports on the Child Behavior Checklist. Epidemiol Psychiatry Sci.

[21] Achenbach TM, Ivanova MY, Rescorla LA, Turner LV, Althoff RR (2016). Internalizing/Externalizing Problems: Review and recommendations for clinical and research applications. J Am Acad Child Adolesc Psychiatry.

[22] Achenbach TM, Dumenci L, Rescorla LA (2003). DSM-oriented and empirically based approaches to constructing scales from the same item pools. J Clin Child Adolesc Psychol.

[23] Biederman J, Ball SW, Monuteaux MC, Kaiser R, Faraone SV (2008). CBCL clinical scales discriminate ADHD youth with structured-interview derived diagnosis of oppositional defiant disorder (ODD). J Atten Disord.

[24] Biederman J, Monuteaux MC, Kendrick E, Klein KL, Faraone SV (2005). The CBCL as a screen for psychiatric comorbidity in paediatric patients with ADHD. Arch Dis Child.

[25] Achenbach TM, Ruffle TM (2000). The Child Behavior Checklist and related forms for assessing behavioral/emotional problems and competencies. Pediatr Rev.

[26] Achenbach TM, Ivanova MY, Rescorla LA (2017). Empirically based assessment and taxonomy of psychopathology for ages 1(1/2)-90 + years: developmental, multi-informant, and multicultural findings. Compr Psychiatry.

[27] Goodman R (1997). The strengths and difficulties questionnaire: a research note. J Child Psychol Psychiatry.

[28] Goodman R (1999). The extended version of the strengths and difficulties questionnaire as a guide to child psychiatric caseness and consequent burden. J Child Psychol Psychiatry.

[29] Goodman R, Meltzer H, Bailey V (1998). The Strengths and Difficulties Questionnaire: a pilot study on the validity of the self-report version. Eur Child Adolesc Psychiatry.

[30] Fleitlich B, Cortázar PG, Goodman R (2000). Questionário de capacidades e dificuldades (SDQ). Infanto rev neuropsiquiatr infanc adolesc.

[31] Saur AM, Loureiro SR (2012). Qualidades psicométricas do Questionário de Capacidades e Dificuldades: revisão da literatura. Estudos de Psicologia (Campinas).

[32] Fleitlich-Bilyk B, Goodman R (2004). Prevalence of child and adolescent psychiatric disorders in southeast Brazil. J Am Acad Child Adolesc Psychiatry.

[33] Goodman A, Fleitlich-Bilyk B, Patel V, Goodman R (2007). Child, family, school and community risk factors for poor mental health in Brazilian schoolchildren. J Am Acad Child Adolesc Psychiatry.

[34] Goodman R, Ford T, Simmons H, Gatward R, Meltzer H (2000). Using the Strengths and Difficulties Questionnaire (SDQ) to screen for child psychiatric disorders in a community sample. Br J Psychiatry.

[35] Cheng S, Keyes KM, Bitfoi A, Carta MG, Koc C, Goelitz D (2018). Understanding parent-teacher agreement of the strengths and difficulties questionnaire (SDQ): comparison across seven European countries. Int J Methods Psychiatr Res.

[36] Raiker JS, Freeman AJ, Perez-Algorta G, Frazier TW, Findling RL, Youngstrom EA (2017). Accuracy of achenbach scales in the screening of attention-deficit/hyperactivity disorder in a community mental health clinic. J Am Acad Child Adolesc Psychiatry.

[37] American Psychiatric Association (2000). Diagnosis and statistics manual of mental disorders [DSM-IV-TM].

[38] Cohen A (2002). Mental health among indigenous population. An international view. Epidemiol Psichiatr Soc.

[39] Lampert TL, Polanczyk G, Tramontina S, Mardini V, Rohde LA (2004). Diagnostic performance of the CBCL-attention problem scale as a screening measure in a sample of Brazilian children with ADHD. J Atten Disord.

[40] Goodman R, Neves dos Santos D, Robatto Nunes AP, Pereira de Miranda D, Fleitlich-Bilyk B, Almeida Filho N (2005). The Ilha de Mare study: a survey of child mental health problems in a predominantly African-Brazilian rural community. Soc Psychiatry Psychiatr Epidemiol.

[41] Rohde LA, Biederman J, Busnello EA, Zimmermann H, Schmitz M, Martins S (1999). ADHD in a school sample of Brazilian adolescents: a study of prevalence, comorbid conditions, and impairments. J Am Acad Child Adolesc Psychiatry.

[42] Vasconcelos MM, Werner J, Malheiros AF, Lima DF, Santos IS, Barbosa JB (2003). Attention deficit/hyperactivity disorder prevalence in an inner city elementary school. Arq Neuropsiquiatr.

[43] Fontana RS, Vasconcelos MM, Werner J, Goes FV, Liberal EF (2007). ADHD prevalence in four Brazilian public schools. Arq Neuropsiquiatr.

[44] Guardiola A, Fuchs FD, Rotta NT (2000). Prevalence of attention-deficit hyperactivity disorders in students. Comparison between DSM-IV and neuropsychological criteria. Arq Neuropsiquiatr.

[45] Anselmi L, Fleitlich-Bilyk B, Menezes AM, Araujo CL, Rohde LA (2010). Prevalence of psychiatric disorders in a Brazilian birth cohort of 11-year-olds. Soc Psychiatry Psychiatr Epidemiol.

[46] Biederman J, Newcorn J, Sprich S (1991). Comorbidity of attention deficit hyperactivity disorder with conduct, depressive, anxiety, and other disorders. Am J Psychiatry.

